# Plasmonic Coupling in Silver Nanoparticle Aggregates and Their Polymer Composite Films for Near*-*Infrared Photothermal Biofilm Eradication

**DOI:** 10.1021/acsanm.1c00668

**Published:** 2021-05-05

**Authors:** Padryk Merkl, Shuzhi Zhou, Apostolos Zaganiaris, Mariam Shahata, Athina Eleftheraki, Thomas Thersleff, Georgios A. Sotiriou

**Affiliations:** † Department of Microbiology, Tumor and Cell Biology, Karolinska Institutet, Stockholm SE-17177, Sweden; ‡ Department of Materials and Environmental Chemistry, Stockholm University, Stockholm 10691, Sweden

**Keywords:** nanoaggregates, on-demand disinfection, Ag, nanosilver, near-IR

## Abstract

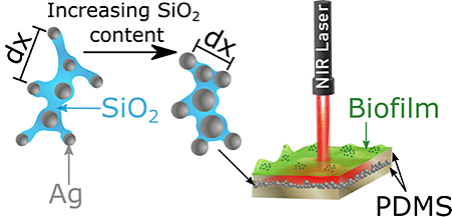

Plasmonic nanoparticles with near-IR (NIR) light absorption are highly attractive in biomedicine for minimally invasive photothermal treatments. However, these optical properties are typically exhibited by plasmonic nanostructures with complex, nonspherical geometries that may prohibit their broad commercialization and further integration into photothermal devices. Herein, we present the single-step aerosol self-assembly of plasmonic nanoaggregates that consisted of spherical silver nanoparticles with tunable extinction from visible to NIR wavelengths. This tunable extinction was achieved by the addition of SiO_2_ during the flame synthesis of the nanoparticles, which acted as a dielectric spacer between the spherical silver nanoparticles and was also computationally validated by simulating the extinction spectra of similar silver nanoaggregates. These plasmonic nanoaggregates were easily deposited on silicone polymeric surfaces and further encased with a top polymer layer, forming plasmonic photothermal nanocomposite films. The photothermal properties of the NIR nanocomposite films were utilized to eradicate the established biofilms of clinically relevant *Escherichia coli* and *Staphylococcus aureus,* with a relationship observed between the final surface temperature and biofilm eradication.

## Introduction

Plasmonic nanomaterials exhibit striking optical properties that render them useful in highly sensitive diagnostic devices, potent medical treatments, and industrial tools.^1,2^ Gold and silver are two of the most well-known plasmonic nanomaterials because they exhibit strong plasmonic properties in the visible region of the electromagnetic spectrum. A common feature of plasmonic materials is their photothermal behavior, which has been applied for targeted, on-demand, and localized heat generation for anticancer and antimicrobial therapies.^3,4^ However, visible light is strongly absorbed by biological tissues, limiting their medical application in vivo. Thus, nanostructures with plasmonic properties in the near-infrared (NIR) regions are an attractive target in biomedicine.^5^

The plasmonic extinction (absorption and scattering) of silver and gold nanoparticles can be shifted into the NIR region by modifying their size and shape. Plasmonic nanoparticles with shapes such as nanoshells,^6^ nanocubes,^7^ nanocages,^8^ nanorods,^9^ nanostars,^10^ nanotriangles,^11^ and nanobipyramids^12^ can be used to tune the maximum extinction wavelength into the NIR region.^13^ However, these complex geometries typically require elaborate synthetic procedures, and their facile, homogeneous, and reproducible deposition on large-scale surfaces is limited by technological hurdles. Even though NIR photothermal silver nanomaterials exist, such as nanotriangles made by one-step seedless method,^14^ advantages of the developed photothermal surfaces here include the utilization of the controlled plasmonic coupling of spherical silver nanoparticles active in the NIR region made by a rapid, scalable, and reproducible nanomanufacturing process,^15^ which allows nanoparticle deposition on a selected substrate without any prior treatment or functionalization.^16^ An alternative approach to extend the extinction of plasmonic nanostructures into the NIR region is to exploit the plasmonic interparticle coupling. This can be achieved by the reciprocal interaction of neighboring, plasmonic, primary nanoparticles with small interparticle distances.^17^ This has been demonstrated by coating plasmonic nanoparticles with a thin SiO_2_ shell that acted as a dielectric spacer, effectively providing a limit on the minimum, interplasmonic, particle distance.^17−20^ Such a hermetic SiO_2_ shell may prevent any potential dissolution of the core nanomaterial^21^ and offers support for further surface functionalization.^22^ Furthermore, use of an inorganic, dielectric spacer inhibits any sintering and restructuring of the metallic plasmonic material that otherwise might occur due to the high temperatures achieved under laser irradiation.^18^

Several applications of NIR-absorbing nanoparticles have been demonstrated, such as the plasmon-enhanced, antioxidant activity of gallic acid by SiO_2_-coated nanosilver^20^ and the photothermal killing of cancer cells and biofilms by NIR-absorbing plasmonic structures.^18,23,24^ Although several previous studies have demonstrated successful killing of planktonic bacteria with nanosilver,^25−27^ established biofilms are rather resilient structures and more difficult to eradicate than planktonic bacteria.^28^ This photothermal effect is a key target application of plasmonic nanoparticles and arises since the scattering of incident light is dominated by nonradiative decay processes, leading to increase in temperature and the photothermal effect.^29^ Although isolated nanoparticles have been well-explored for therapeutic applications, the facile synthesis of spatially homogeneous, NIR-absorbing, plasmonic films and coatings on medical devices remains an underexplored topic. The application of films on medical devices could allow the localized, on-demand, photothermal killing of bacterial biofilms that form on the device surfaces^30^ and are a frequent cause of infection.^31,32^ To facilitate potential commercialization and broad employment, NIR, photothermal coatings would ideally consist of inexpensive materials (e.g., silver instead of gold) and nanospheres that are easy to make instead of more complex geometries.

In this work, we present a facile strategy to produce NIR, photothermal, plasmonic films based on fractal-like nanoaggregates that consisted of spherical silver nanoparticles and a dielectric spacer to tune the interparticle distance and, thus, the plasmonic coupling. The as-prepared plasmonic nanoaggregates were deposited on selected substrates by aerosol self-assembly during their flame synthesis. We systematically studied the effect of the dielectric spacer content on the extinction of the plasmonic films and their NIR photothermal response. Photothermal films were fully encased within a thin polymer layer to allow for the fabrication of photothermal, polymer coatings on medical devices. As a proof of concept, we grew mature *Staphylococcus aureus* and *Escherichia coli* biofilms from clinically relevant strains on the surface of such films and quantified the biofilm eradication upon NIR laser irradiation by evaluating the number of viable bacteria retrieved from the biofilm after irradiation. This work lays the foundation for the development of plasmonic NIR photothermal films utilizing inexpensive spherical silver nanoparticles for the biomedical applications.

## Results and Discussion

### NIR Plasmonic Nanoparticle Films

**Figure 1:**
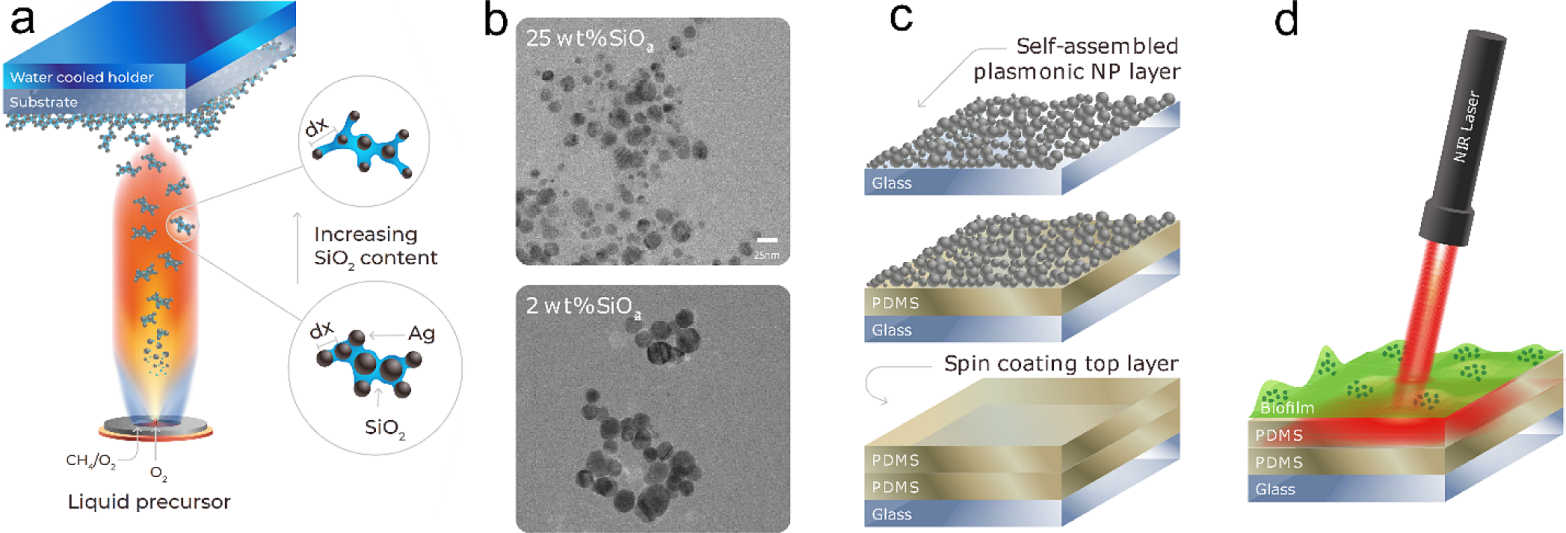
(a) Schematic of the flame aerosol deposition process. Controlling the composition of the liquid precursor solution dictated the final morphology of the as-prepared Ag/SiO_2_ nanoaggregates; increasing SiO_2_ content (that acts as a dielectric spacer) affected the silver interparticle distance (d*x*: average interparticle  distance). (b) TEM images of particles recovered from films deposited with a nominal SiO_2_ content of 25 and 2 wt %, demonstrating the difference in the silver interparticle distance. Scale bar is the same in both images. (c) Schematic of the three different films synthesized: uncoated film deposited on glass (top), PDMS-coated (middle) film, and PDMS-encased (bottom) film. (d) Schematic representation summarizing the antibiofilm mechanism of the developed films: The PDMS-encased plasmonic nanoparticles converted 808 nm NIR laser light into heat to effectively remove the biofilm that formed on the surface of the simulated medical device.

Plasmonic nanoparticle films were deposited onto glass substrates by positioning a water-cooled holder above the flame, causing thermophoretic deposition of nanoaggregates onto the substrates, as shown in Figure 1a. The particles consisted of both silver and nanostructured SiO_2_ support that acted as a dielectric spacer for the individual spherical silver nanoparticles, as shown in the transmission electron microscopy (TEM) images in Figure 1b. The nanoaggregates with high dielectric spacer contents (25 wt % SiO_2_) consisted of smaller silver particles with larger interparticle distances (d*x:* average interparticle  distance) than those with low dielectric spacer contents (2 wt % SiO_2_). The nanoparticle films were either deposited directly on the glass, to allow for their optical and thermal characterization (Figure 1c), or on a polydimethylsiloxane (PDMS)-coated glass substrate, on top of which a second PDMS layer was subsequently spin-coated to generate a PDMS-encased nanoparticle layer. The porous structure of the three-dimensional plasmonic film facilitated the infusion of the second PDMS layer.^16^ This action was taken to avoid the undesirable release of silver nanoparticles or ions and to simulate the surface of a medical device (e.g., a catheter or a wound mesh) for their further investigation as antibiofilm surfaces (Figure 1d).

**Figure 2:**
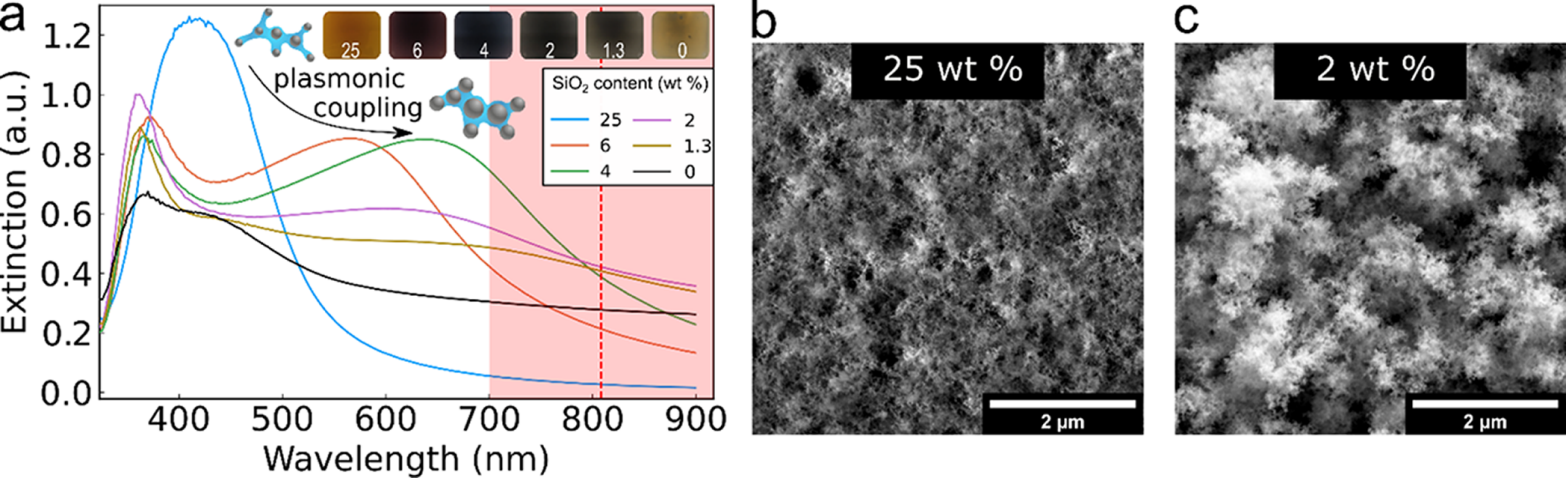
(a) UV–vis spectra of plasmonic nanoaggregates deposited on glass slides, demonstrating the change in absorption properties with the decrease in the dielectric spacer SiO_2_ content. Inset shows digital photographs taken fromthe glass slides (SiO_2_ content from left to right: 0, 1.3, 2, 4, 6, and 25 wt %). Film deposition times used here were 20 s to allow for accurate spectrophotometric measurements. (b) and (c) Top-view SEM images of nanoaggregate films with 25 and 2 wt % SiO_2_ content, respectively.

The optical properties of nanoparticle films made with varying dielectric spacer contents are shown in Figure 2a, where a decreasing content caused an increase in the NIR extinction (indicated by the red shading from 700 nm). Silver particles synthesized without any spacer (0 wt % SiO_2_) showed poor colloidal stability (Figure S1b) and lower extinction than nanoaggregates with low dielectric spacer contents. These spectra also qualitatively agreed with the inset of Figure 2a, where images of the glass substrates coated with the silver nanoparticles are shown. The classical yellow color of the silver nanoparticles was observed at a high dielectric spacer content (25 wt % SiO_2_), indicating that minimal interparticle coupling occurred among the spherical, silver nanoparticles. However, upon decreasing the dielectric spacer content to 6 wt % SiO_2_, the films acquired a dark red tint, and the extinction broadened to cover a large part of the visible spectrum; upon the further decrease of the dielectric spacer content from 4 to 1.3 wt %, the films appeared black. These spectra were determined from films deposited for only 20 s to avoid high extinction values above the detection limit of the instrument (Figure S2a). The nanoparticle film thickness along with their optical and photothermal properties are tuned by the deposition duration during their self-assembly (Figure S3a). However, all further characterization was performed with nanoparticle films deposited for 80 s. The dashed, red, vertical line in Figure 2a identifies the laser wavelength of 808 nm, often used for NIR, photothermal treatment, which was also used in this study. Figure 2b,c shows the top-view scanning electron microscopy (SEM) images of films with a high dielectric spacer content (25 wt %) and with a low content (2 wt %), respectively, both of which exhibited a rather porous structure.

**Figure 3:**
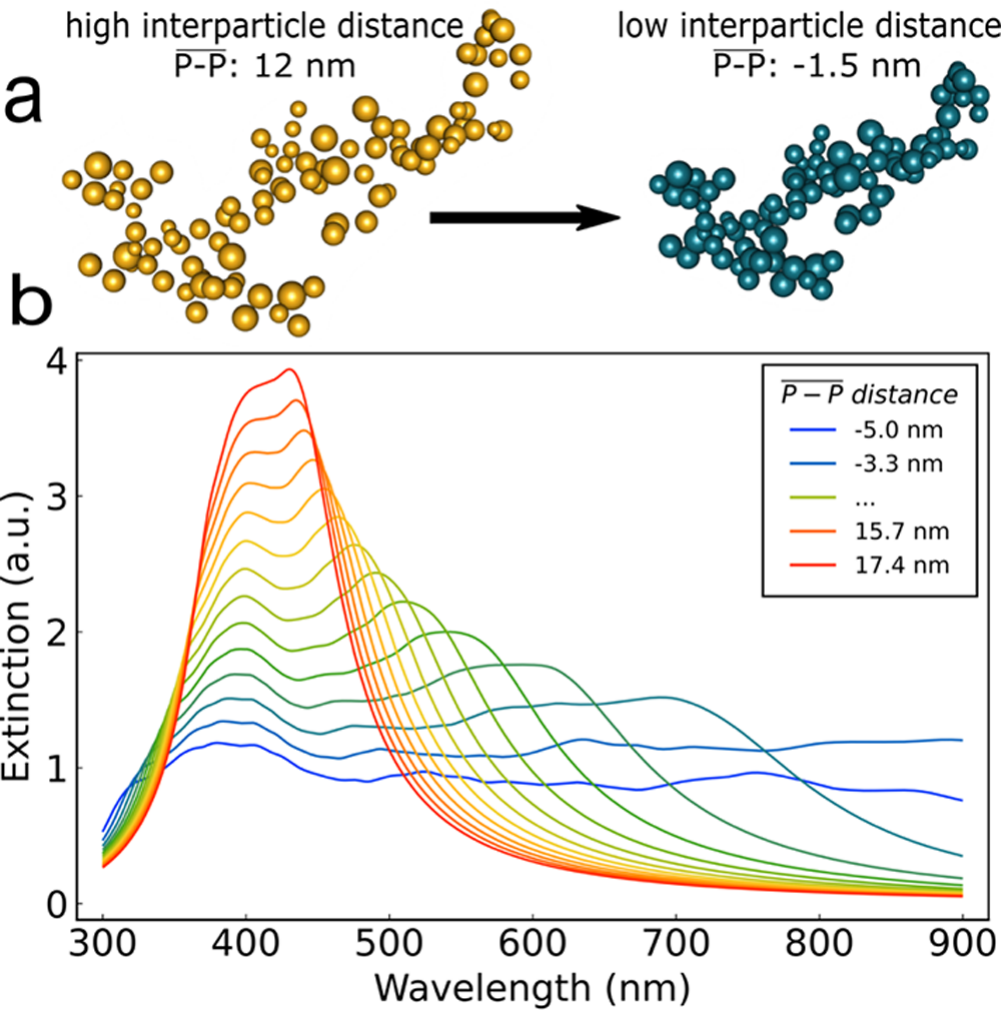
(a) Simulated nanoaggregates with high interparticle distance ( distance) (yellow, left side) and low distance (green, right side) represented by the differences in interparticle spacing. A negative  distance indicates that the particles are partially sintered. (b) Simulated extinction of fractal-like nanoparticle agglomerates with varying interparticle distance (average  distance), generated by multiplying the positions of the primary particles of agglomerates centered around zero by a factor.

To better understand the plasmonic interparticle coupling that resulted in an NIR extinction increase with the decrease in SiO_2_ content, simulations were performed by generating fractal-like, polydisperse aggregates using FracVAL.^33^ The extinction of these aggregates, which contained only silver nanoparticles (0 wt % SiO_2_), was simulated using the coupled dipole approximation of Auguié et al.^34,35^ The primary particles in the as-generated aggregates from FracVAL were nonoverlapping; to generate particle agglomerates with varying interparticle spacings, a multiplication factor was applied in a spherically symmetric fashion about the geometric center of the aggregates, resulting in structures with tunable interparticle distances ( distance) (Figure 3a). The resulting NIR extinctions (Figure 3b) showed a gradual increase with the decrease in the interparticle spacing (negative  distance indicates that spheres are partially sintered) of the generated fractal-like silver nanoaggregates with constant geometric mean primary particle size of 16 nm, geometric standard deviation of 1.2, and 100 primary particles. The resulting spectra were in qualitative agreement with the measured spectra in Figure 2a. The number of particles per agglomerate, fractal dimension (Df), and primary particle size were also varied (simulated extinctions are shown in Figure S4). However, these parameters did not exhibit a strong increase in NIR extinction at relevant values as compared to the interparticle distance.

**Figure 4:**
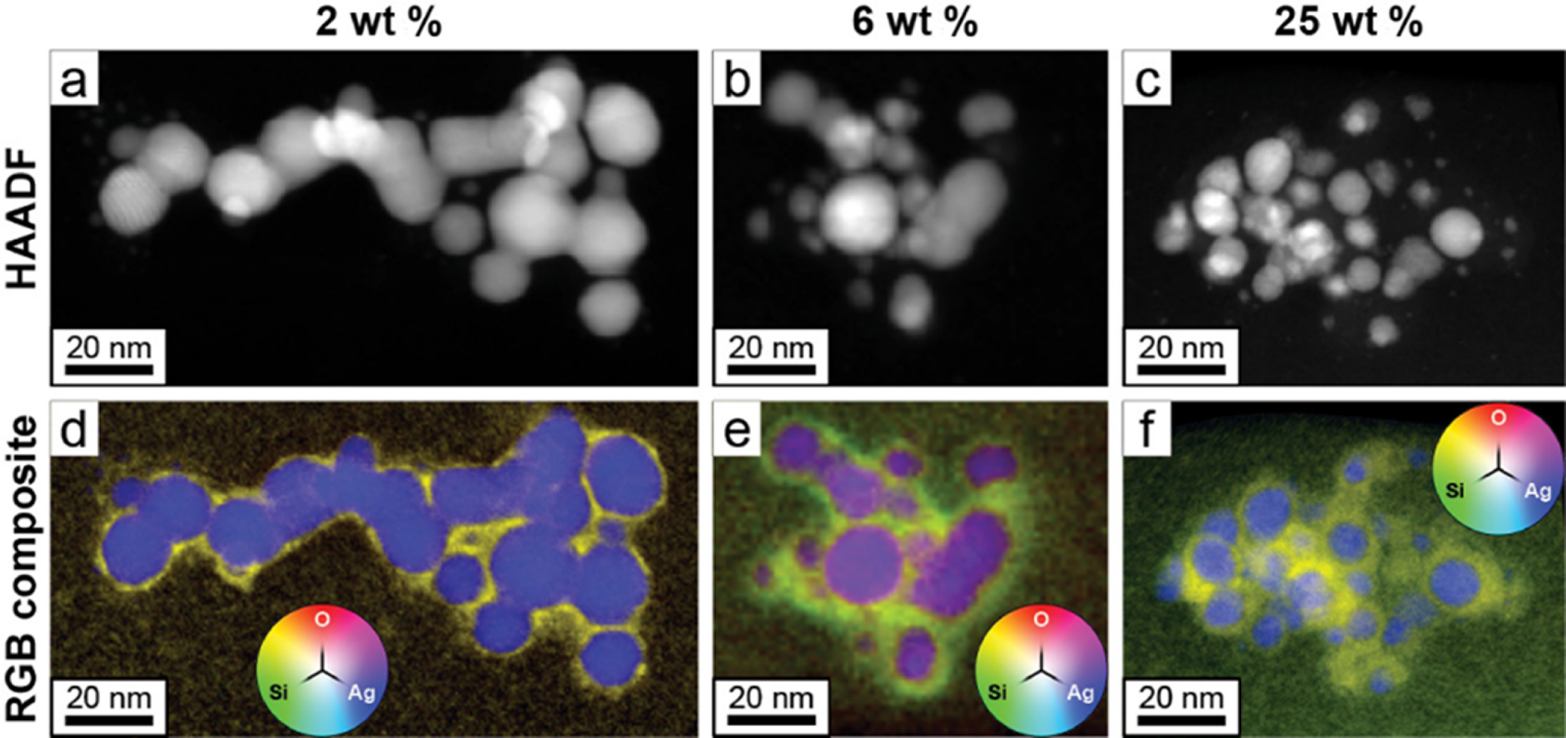
HAADF images of single nanoparticle aggregates for 2, 6, and 25 wt % SiO_2_ (a–c, respectively), and EDX/EELS fusion images with false color showing the elemental composition (blue, Ag; red, O; and green, Si) of the same nanoaggregates for 2, 6, and 25 wt % SiO_2_ (d–f).

Therefore, the increase in the NIR extinction with the decrease in the dielectric spacer content was attributed to the smaller interparticle distances between the spherical silver nanoparticles within the individual nanoaggregates. The nanoscale chemistry and morphology of the dielectric spacers were further assessed in an aberration-corrected TEM using electron energy-loss spectroscopy (EELS) and energy dispersive X-ray spectroscopy (EDX), and these results are summarized in Figure 4. Figure 4a–c presents micrographs from nanoparticle films containing 2, 6, and 25 wt % dielectric spacer, respectively. These images were acquired with TEM in scanning mode using a high-angle annular dark-field (HAADF) detector and therefore dominantly show *Z* contrast. Figure 4d–f present red–green–blue (RGB) composite images representing the relative abundance of O (red), Si (green), and Ag (blue) from the same areas for the same set of dielectric spacers. In each case, the silver nanoparticles are embedded in Si- and O-rich materials, which are present both as a separation between silver nanospheres and as an encapsulant around the nanoaggregates. This is interpreted as the proposed SiO_2_ dielectric spacer, which controls the optical extinction, and the clear increase in interparticle SiO_2_ from 2 to 25 wt % SiO_2_ associates with the increase in NIR extinction. The data for these composite images were extracted from an unsupervised empirical model derived from the combination of three simultaneously acquired hyperspectral datacubes containing low-loss EELS, core-loss EELS, and EDX. These three data sets were combined using a technique known as data fusion prior to variance consolidation via orthogonalization of the joint covariance matrix, as described by Thersleff et al.^36,37^ More details on the analysis and the compositional maps (Figure S5) without treatment are provided in the Supporting Information.

This was further verified by TEM images for all samples (Figure S1a), where the amorphous nanostructured SiO_2_ dielectric spacer was observed that separated the spherical silver nanoparticles within each aggregate and prevented them from coalescing during their flame synthesis. To validate that the NIR extinction originated from the controlled, plasmonic coupling of the silver nanoparticles within the individual aggregates and is not a result of their aerosol deposition as films, the optical properties of colloidal suspensions in ethanol were also studied showing similar behavior as the deposited films (Figure S1b), in agreement with the literature.^38,39^ No significant change in the size distribution of the colloidal suspensions was observed with different SiO_2_ contents (Figure S1c). The amorphous SiO_2_ serves a dual function: (i) keeps the individual silver nanospheres together in each nanoaggregate and (ii) acts as a dielectric spacer to fine-tune the interparticle distance in a controlled manner. These results demonstrated that the NIR extinction is not only a property of the deposited films but also of the fractal-like nanoaggregates themselves. It should be noted that the extinction of the as-prepared nanoaggregates was not expected to be significantly affected by the silver nanoparticle size here, as their average crystal size showed small changes of less than 1 nm from 2 to 6 wt % SiO_2_ contents (Figure S2c,d). The TEM size distributions also showed minor decreases in geometric mean diameter of less than 3 nm (less than 20% change) between 2 and 6 wt % SiO_2_ (Figure S6). Measurements of the interparticle distances from TEM images were also performed demonstrating an increase in interparticle distance of 2 nm (more than 60% change) between 2 and 6 wt % SiO_2_ (Figure S8). Side-view images of the films (Figure S9a) showed similar morphologies and thicknesses (Figure S9b) between 1.3 and 6 wt % SiO_2_ content, but the film with 25 wt % was thicker and qualitatively denser.

**Figure 5:**
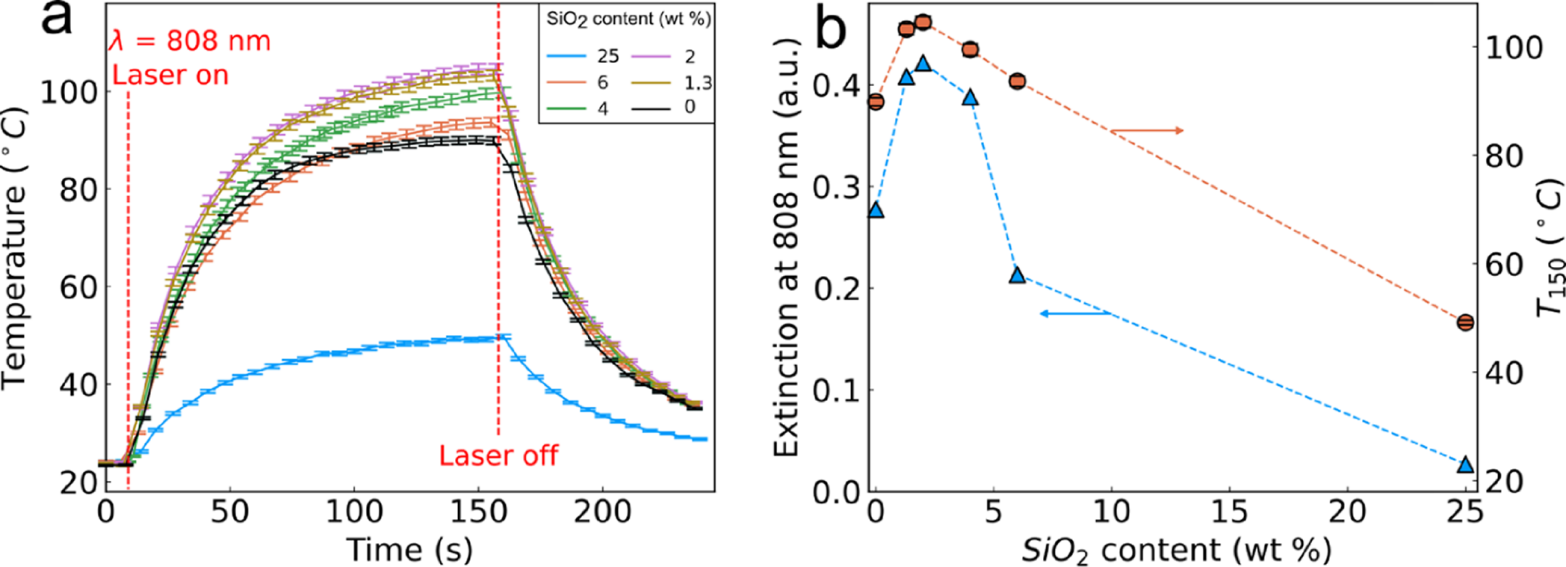
(a) Temperature evolution over time measured by an IR camera while irradiating the sample with 808 nm laser light at an intensity of 1 W/cm^2^. (b) Extinction of films at 808 nm (blue triangles, the left-hand *y* axis) and temperature recorded after 150 s of irradiation with 808 nm laser light (orange circles, right-hand *y* axis) as a function of the dielectric spacer SiO_2_ content.

The temperatures attained by the nanoparticle films during laser irradiation (1 W/cm^2^, λ = 808 nm) measured by an IR thermal camera are shown as a function of time in Figure 5a, where a rapid increase was observed that subsequently flattened as the substrates approached equilibrium. As soon as the laser was switched off, the films began to cool, demonstrating the high level of thermal control of these plasmonic photothermal films. Figure 5b shows the maximum extinction values at 808 nm (blue triangles, left axis) and maximum temperatures reached after 150 s of irradiation (red circles, right axis) as a function of the dielectric spacer SiO_2_ content. There was a strong correlation between the extinction at 808 nm and the final temperature (Figure S2b). The highest extinction at 808 nm and temperature after 150 s was achieved for the 2 wt % dielectric spacer content, and this sample was chosen for further experiments.

### Polymer Nanocomposite Fabrication by PDMS Coating

**Figure 6:**
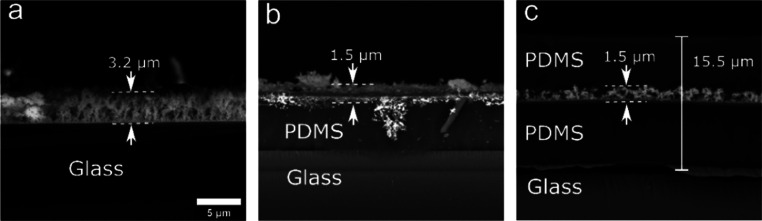
Side-view SEM images of the deposited films (*t*_D_ = 80 s) showing (a) nanoaggregate films deposited on glass, (b) films deposited on a PDMS-coated glass, and (c) films encased in PDSM by an additional layer by spin coating. Scale bar is the same in all images.

Direct flame aerosol deposition allows for nanoparticle film fabrication on both inorganic and polymer surfaces.^16,40^ To evaluate whether the plasmonic films retained their optical properties when deposited on a polymer surface, a 6 μm PDMS layer was spin-coated onto the glass substrates, and the fractal-like, plasmonic, nanoaggregate film was subsequently deposited. The nanoparticle film was then encased within PDMS by spin-coating an additional layer of equal thickness. Upon SEM examination of the cross sections of the three different nanoparticle films (deposited on glass, deposited on PDMS-coated glass, encased within two PDMS layers, Figure 6), it was observed that the nanoparticle films on the PDMS-coated glass were thinner than those deposited on glass for the same deposition duration (*t*_D_ = 80 s). This was attributed to the different thermal conductivity of PDMS compared to glass, which affected the thermophoretic deposition. The spectra after the PDMS encasing exhibit some changes in their peak extinction due to film restructuring (Figure S10); however, the PDMS-encased nanoparticle film retains the spatial homogeneity and NIR extinction of the bare plasmonic film on glass, while exhibiting a superior structural stability. The photothermal efficiency of the nanoparticle films was determined to be 50% according to the analysis described by Breitenborn et al.^41^ (Figure S11), and the deposited mass was calculated to be 15.4 μg. The developed photothermal coatings exhibit high stability upon repeated treatments (Figure S12). Moreover, silver ion selective measurements of phosphate buffered saline (PBS) incubated for 24 h at 37 °C with the PDMS-encased films did not detect any silver ions. The PDMS-encased plasmonic films were therefore sealed while retaining their NIR optical extinction and leaving their photothermal properties unaltered after immersion in aqueous solutions for 24 h at 60 °C.

### On-Demand Eradication of Bacterial Biofilms

**Figure 7:**
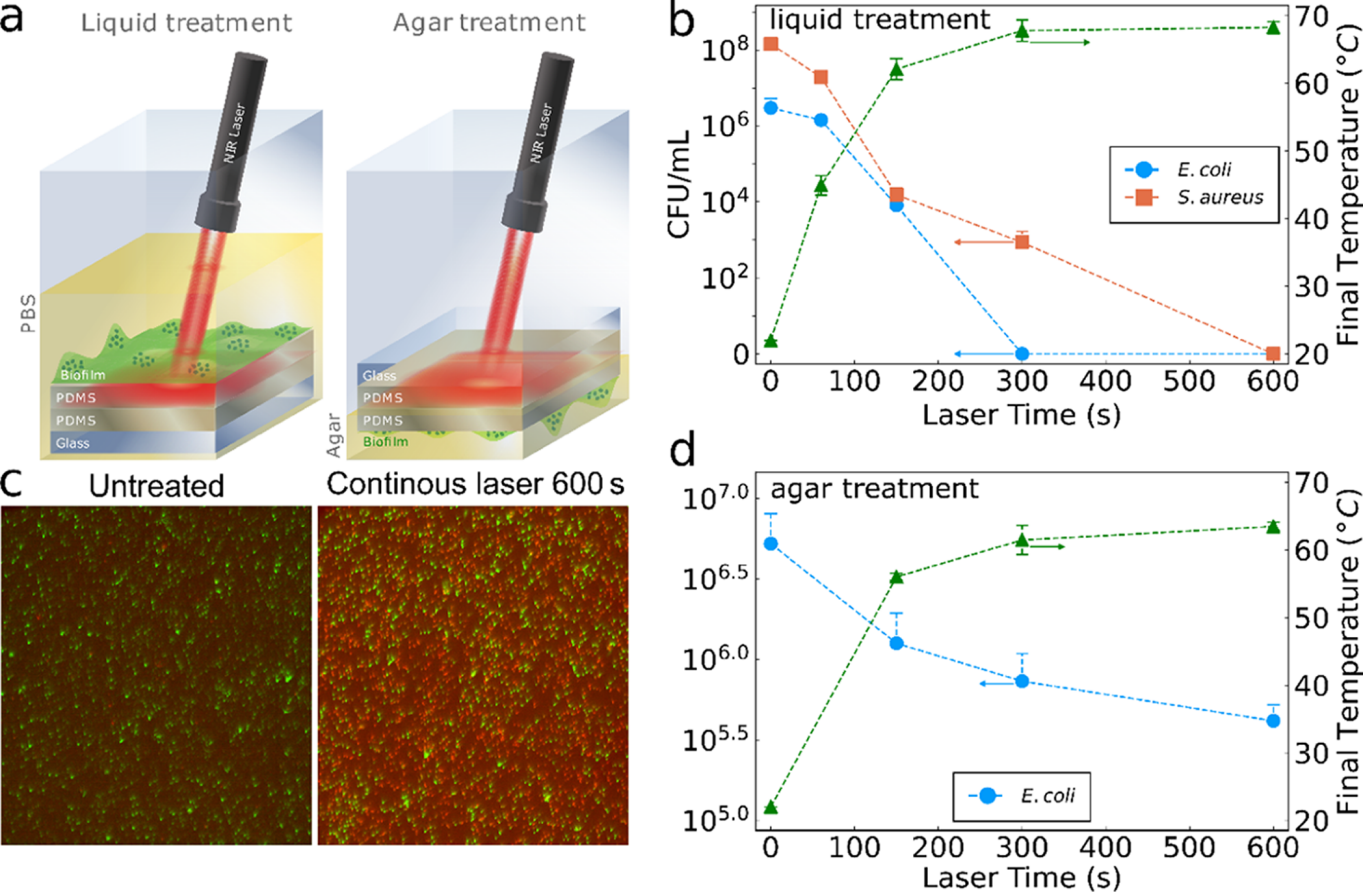
(a) Schematic depiction of the two biofilm treatment models used, either irradiating the polymer films with established biofilm on their surface when immersed in liquid (PBS) or with the biofilm in contact with agar. (b) Bacteria quantification (CFU/mL) of *E. coli* (blue circles) and *S. aureus* (orange squares) biofilms after continuous NIR laser irradiation performed in liquid and the corresponding temperatures (green triangles, right axis). (c) A representative fluorescence image of live/dead staining performed on untreated or 600-s laser-irradiated biofilms. Additional images at identical imaging conditions are shown in Figure S16. (d) Bacteria quantification (CFU/mL) of *E. coli* (blue circles, left axis) and final temperature (green triangles, right axis) as a function of laser time performed on agar plates with biofilm touching the agar and laser illumination from the top. *n* = 3 for all graphs.

The PDMS-encased plasmonic photothermal film was further investigated as an on-demand, antibiofilm surface. Typically, the antibiofilm properties of photothermal surfaces are examined as biofilm inhibition from planktonic bacteria, while here we study the photothermal eradication of the established biofilms, i.e., treatment after the biofilm has already formed that resembles a more clinically relevant scenario. The top PDMS layer simulated the surface of a polymer-based medical device (e.g., catheter, surgical mesh, or wound dressing). The PDMS-encased film was fully immersed in culture medium with either *S. aureus* or *E. coli* for 24 h and subsequently washed to remove planktonic bacteria, leaving the formed established biofilm on its surface. Two different biofilm treatment models were explored, as shown in Figure 7a. In the first model, to simulate the medical device–liquid interface, the encased film with the established biofilm on its surface was submerged in sterile PBS and irradiated (λ = 808 nm, 1.4 W/cm^2^). In the second model, the PDMS-encased film with the established biofilm on its surface was flipped and placed on agar before laser irradiation treatment, simulating the device–tissue interface. During all laser irradiation treatments, the temperature was monitored in real time with a thermal camera to establish the temperature-dependent biofilm eradication process.

Figure 7b shows the colony-forming units (CFU/mL, left axis) retrieved from substrates on which *E. coli* (blue circles) and *S. aureus* (orange squares) were grown and the temperatures reached at the end of laser irradiation for each time point (triangles, right axis) when performed according to the first model. The temperature increase during the biofilm eradication experiments follows the same kinetics as shown in Figure 5a. Continuous laser irradiation of the photothermal coating completely eradicated *E. coli* biofilms after only 300 s, while 600 s was needed for complete eradication of *S. aureus*. The right axis of Figure 7b shows the temperature of the PBS attained at the end of the laser irradiation, showing a clear trend of increased eradication at higher temperatures. The complete eradication of *S. aureus* biofilms upon laser irradiation in air at different intensities was also demonstrated (Figure S13a). This biofilm eradication was also qualitatively confirmed by performing live/dead staining on *E. coli* biofilms on substrates exposed to laser irradiation for 600 s, as shown in Figure 7c, where the bacteria were labeled green if they are alive and red if they are dead. The untreated biofilm exhibited mostly green signals verifying the presence of live bacteria. In contrast, the laser-irradiated sample exhibited mostly red signals, indicating that most bacteria had been eradicated.

To investigate the potential of pulsed laser treatment, six different pulsed laser conditions in the liquid culture setup were assessed with varying on/off times (Figure S14a). The pulses were designed such that the total energy delivered to the sample was 210 J/cm^2^ for all pulsed conditions (corresponding to 150 s of continuous laser irradiation at 1.4 W/cm^2^); therefore, the total treatment durations for the different pulsing conditions were different. The resulting final temperature and CFU/mL values for *E. coli* for each condition (Figure S14b) indicate no additional effect from the pulsed laser treatment, and the eradication was again correlated with the final temperature. Pulsed laser irradiation at varying laser intensities was also shown to successfully eradicate biofilms when performed in air (Figure S13b).

Finally, the effect of laser on the device–tissue model is shown in Figure 7d, where the CFU/mL values (left axis) and final temperature (right axis) of *E. coli* are plotted as a function of the irradiation time. Although the overall degree of eradication observed was significantly less than that in the PBS-immersed configuration, there was a clear trend of a higher antibiofilm effect for a higher final temperature, suggesting that temperature was the most important parameter. This is further validated by ex situ experiments from substrates immersed in liquid and heated using an external heat source to mimic the temperatures induced by NIR irradiation (Figure S15). Nonetheless, the photothermal, plasmonic surfaces were effective upon 808 nm irradiation (1.4 W/cm^2^), with the complete eradication of the established *E. coli* and *S. aureus* biofilms treated for 300 and 600 s, respectively. Only planktonic bacterial eradication with photothermal silver-based nanoparticles in the NIR region had been achieved prior to this report. Fang et al.^42^ reported on core/shell, Fe_3_O_4_-coated, silver nanoparticles with the complete eradication of planktonic *E. coli* and *S. aureus* under 600 s irradiation at 2 W/cm^2^, while Fan et al.^25^ reported silver nanotriangles that eradicated 98% of planktonic *Enterococcus faecalis* at an intensity of 2 W/cm^2^. A more detailed comparison is provided in Table S1. The complete eradication of the established bacterial biofilms instead of the planktonic bacteria utilizing spherical, silver nanoaggregates and low laser intensities in a short time frame highlighted the potential of the photothermal films developed herein.

An important aspect in the successful application of photothermal films for treating medical-device-related infections is the impact that the required irradiation and temperatures for biofilm eradication may have on the healthy tissue of the patient. Exposure of tissue to temperatures above 45 °C for an hour can lead to cell death; and therefore, careful control of temperatures in the tissues surrounding the device is required.^3^ Due to the on-demand nature of the developed photothermal surface and the development of sophisticated noninvasive NIR methods of thermometry,^43,44^ the developed films could be carefully controlled to avoid delivering harmful temperatures and the irradiation intensity optimized to conform to international standards for safe use of lasers. These considerations highlight the need for further in vivo antibiofilm experiments. Due to the on-demand nature of the developed photothermal surface and the development of sophisticated noninvasive NIR methods of thermometry,^43,44^ the developed films could be carefully controlled to avoid delivering harmful temperatures.

## Conclusions

In this work, we demonstrated the fabrication of NIR, photothermal, plasmonic films based on nanoaggregates that consisted of spherical silver nanoparticles. The desired NIR photothermal properties were achieved by exploiting the controlled plasmonic coupling among the spherical silver nanoparticles within each nanoaggregate by the addition of a dielectric spacer during the nanoparticle flame synthesis, which finely tuned the plasmonic interparticle distance. The as-prepared nanoaggregates were deposited on a large scale (cm^2^) on selected substrates by aerosol self-assembly and were subsequently encased within a thin polymer layer to form polymer nanocomposite films. The NIR photothermal properties were retained in these plasmonic nanocomposite films, and their potential as a functional coating on medical devices is demonstrated for the triggered, on-demand eradication of biofilms. This work advances the knowledge and understanding in the controlled plasmonic coupling of inexpensive spherical silver nanoparticles and lays the foundation for their utilization as NIR photothermal plasmonic materials, providing the field with an alternative to the otherwise necessary nanostructures with complex geometries that impede their broad commercialization.

## Materials and Methods

Synthesis of plasmonic nanoparticle films was achieved using flame spray pyrolysis.^45^ Silver acetate (99%, Alfa Aesar) was dissolved at 90 °C under reflux in equal proportions of acetonitrile (≥99.5%, Sigma-Aldrich) and 2-ethylhexanoic acid (99%, Sigma-Aldrich) to yield a 0.4 M solution. Hexamethyldisiloxane (≥98%, Sigma-Aldrich) was then added to achieve the desired SiO_2_ wt % in the final Ag/SiO_2_ nanoparticle. The precursor solution was then fed through a capillary at a rate of 5 mL/min by a syringe pump (New Era Pump Systems, Inc.) and dispersed by 5 L/min of oxygen (>99.5%, Strandmöllen AB) with a pressure drop of 1.8 bar. A pilot flame of premixed oxygen/methane (>99.5%, AGA Gas AB) at flow rates of 3.2 and 1.5 L/min, respectively, ignited the spray. Nanoparticle films were deposited on glass slides (76 × 52 × 1 mm^3^, Marienfeld) attached to a water-cooled (16 °C) holder at a height of 22 cm above the burner for 80 s unless otherwise stated.

Spin coating was performed with a 10:1:12 weight mixture of sylgard 184 (Dow Chemicals)/curing agent (Dow Chemicals)/cyclohexane (ACS reagent grade, VWR). Spin coating was performed on the glass substrates with a WS-650MZ-23NPPB spin coater (Laurell), and an initial spin speed of 500 rpm was used for 10 s, which was subsequently accelerated to 4000 rpm at an acceleration of 2000 rpm/s for 50 s. The films were subsequently cured at 60 °C for 48 h.

Laser irradiation was accomplished with an 808 nm fiber-coupled diode laser (Laser Century) passed through a collimator and subsequently a custom 3D-printed optical shutter and top-hat diffuser with a square output profile. The laser power was measured using an S425C thermal optical power meter (Thorlabs) after the diffuser and the diffuser height was set to achieve the desired beam intensity at the desired irradiation plane. The temperature was monitored using an 871 thermal imaging camera (testo). TEM measurements were performed on copper grids dipped into particle dispersions prepared by sonicating small fractions of coating in ethanol. The TEM images were collected with a Talos 120C G2 with Ceta-D detector and 120 kV LaB6 source. Size distributions from TEM were obtained by drawing circular regions of interest around primary particles in ImageJ and exporting the measured values for analysis; the TEM images used and particles counted can be found in Figure S7. Interparticle distances were assessed by drawing circular regions of interest around primary particles in ImageJ and exporting the regions of interest, and the interparticle distance was then estimated by calculating the mean distance between a circle and the two closest neighboring circles based on the center point and diameter of each measured circle. The images used can be found in Figure S1. Due to the 3D aggregate nature of the particles and 2D nature of the TEM images, the values obtained should be used only for qualitative comparison. Dynamic light scattering (Zetasizer Ultra, Malvern Panalytical) and ultraviolet–visible (UV–vis) measurements on colloidal suspensions were performed by retrieving coating from glass substrates by sonication in ultrapure water for 10 min.

Bacterial experiments were performed with *S. aureus* (ATCC 25 923) and *E. coli* (HVM52, isolated from a urinary catheter). *E. coli* and *S. aureus* were cultured in lysogeny broth, and overnight liquid cultures were prepared from frozen stock and subsequently diluted to an optical density of 0.1. The diluted stock (300 μL) was added into 48-well plates (Costar) to which test substrates were added with the coated side upwards and allowed to incubate for 24 h. The substrates were removed, washed three times by submersion in PBS to remove planktonic bacteria, and placed in a new well of a 48-well plate with 300 μL of PBS or inverted and placed gently on the surface of lysogeny agar, as shown in Figure 6a. The substrates were subjected to laser irradiation at an intensity of 1.4 W/cm^2^ for variable times, after which the temperature was recorded and the substrates placed in a 2 mL Eppendorf tube containing PBS. The tubes were vortexed for 30 s, sonicated in a bath sonicator (VWR) for 1 min, and subsequently vortexed for a further 30 s to retrieve the biofilm-forming bacteria from the substrate according to the protocol developed by Mandakhalikar et al.^46^ From the liquid in the tubes, appropriate dilutions were prepared and 100 μL was withdrawn and spread onto lysogeny agar plates for colonies to be counted the next day. The effect of temperature was further assessed by treating substrates with biofilms of *E. coli* HVM52 in a heat block set such that the liquid temperature reached similar temperatures for similar times to mimic irradiation: 45, 62, and 68 °C after 90, 150, and 300 s, respectively. Subsequent CFU quantification was performed as mentioned above. Fluorescence microscopy was performed with a Deltavision elite inverted microscope (Applied Precision) equipped with a 20× air objective (Zeiss NA 0.45) on biofilms stained with live–dead stain (Thermofischer) using FITC and TRITC filter sets for live and dead signals, respectively. All images were collected at identical imaging conditions and contrast/brightness adjusted identically. Silver ion measurements were performed on PBS incubated for 24 h at 37 °C with PDMS encased films with a silver ion-selective electrode (Mettler Toledo) attached to an ion meter (SevenCompact, Mettler Toledo).

Simulations of the plasmonic behavior of aggregates were performed using the plasmonic coupled dipole approximation implemented by Auguié^34^ applied to fractal-like polydisperse nanoparticle agglomerates generated using FracVal.^33^ Unless otherwise specified, the fractal dimension used was 1.8, the fractal prefactor was 1.3, the geometric mean size was 16 nm with a geometric standard deviation of 1.2, and 100 primary particles were used per nanoaggregate. Each extinction spectrum shown is the mean of 20 different aggregates. For a full list of input parameters and the additional code used, please see the Supporting Information.

The data in Figure 4 were acquired on a double-aberration corrected Themis Z TEM (Thermo Fischer) operated in scanning mode at 300 kV. Aberrations were corrected up to fifth order, and a focused electron probe was scanned across the agglomerates pictured in the figure with a dwell time of approximately 2.5 ms, a convergence angle of 21.5 mrad, and a probe current of 500 pA. A Super-X EDX detector was used to collect the X-ray emission (EDX), while a Quantum Gatan Image Filter operating in dual-EELS mode was used to disperse all of the transmitted electrons scattered through angles smaller than 23 mrad. In this way, both core-loss and low-loss hyperspectral datacubes were collected in tandem with the EDX data. Additional details of the analysis can be found in the Supporting Information with the raw data used in Figure S5.

## Supporting Information Available

Additional film characterization (TEMs, XRD patterns, SEMs, and simulated extinction) and their analyses are provided in the supporting information (PDF). R code used for generation of nanoparticle aggregates by FracVAL and evaluation of their plasmonic absorbance properties are available for free on GitHub https://github.com/padmer/FracVAL_cda_helpers

## Abbreviations

NIR: near infrared
PBS: phosphate buffered saline
TEM: transmission electron microscopy
XRD: powder X-ray diffraction
SEM: scanning electron microscopy
PDMS: polydimethylsiloxane
CFU: colony-forming units
Df: fractal dimension
