# Supplementary material for: Plasmonic Coupling in Silver Nanoparticle Aggregates and Their Polymer Composite Films for Near-Infrared Photothermal Biofilm Eradication
Source: ACS Appl Nano Mater. 2021 May 5;4(5):5330–9. doi: 10.1021/acsanm.1c00668 (PMC8165696; doi:10.1021/acsanm.1c00668)
Supplement: Supplementary file 1 — an1c00668_si_001.pdf [file an1c00668_si_001.pdf]

SUPPORTING INFORMATION

Plasmonic Coupling in Silver Nanoparticle Aggregates and their Polymer Composite Films for Near-Infrared Photothermal Biofilm Eradication

Padryk Merkl[a], *Shuzhi Zhou*[a], *Apostolos Zaganiaris*[a], *Mariam Shahata*[a], *Athina Eleftheraki*[a], *Thomas Thersleff*[b] *and Georgios A. Sotiriou*[a*]

[a] Department of Microbiology, Tumor and Cell Biology, Karolinska Institutet, SE-17177 Stockholm, Sweden.

[b] Stockholm University, Department of Materials and Environmental Chemistry, Stockholm 10691, Sweden

*Corresponding author: georgios.sotiriou@ki.se

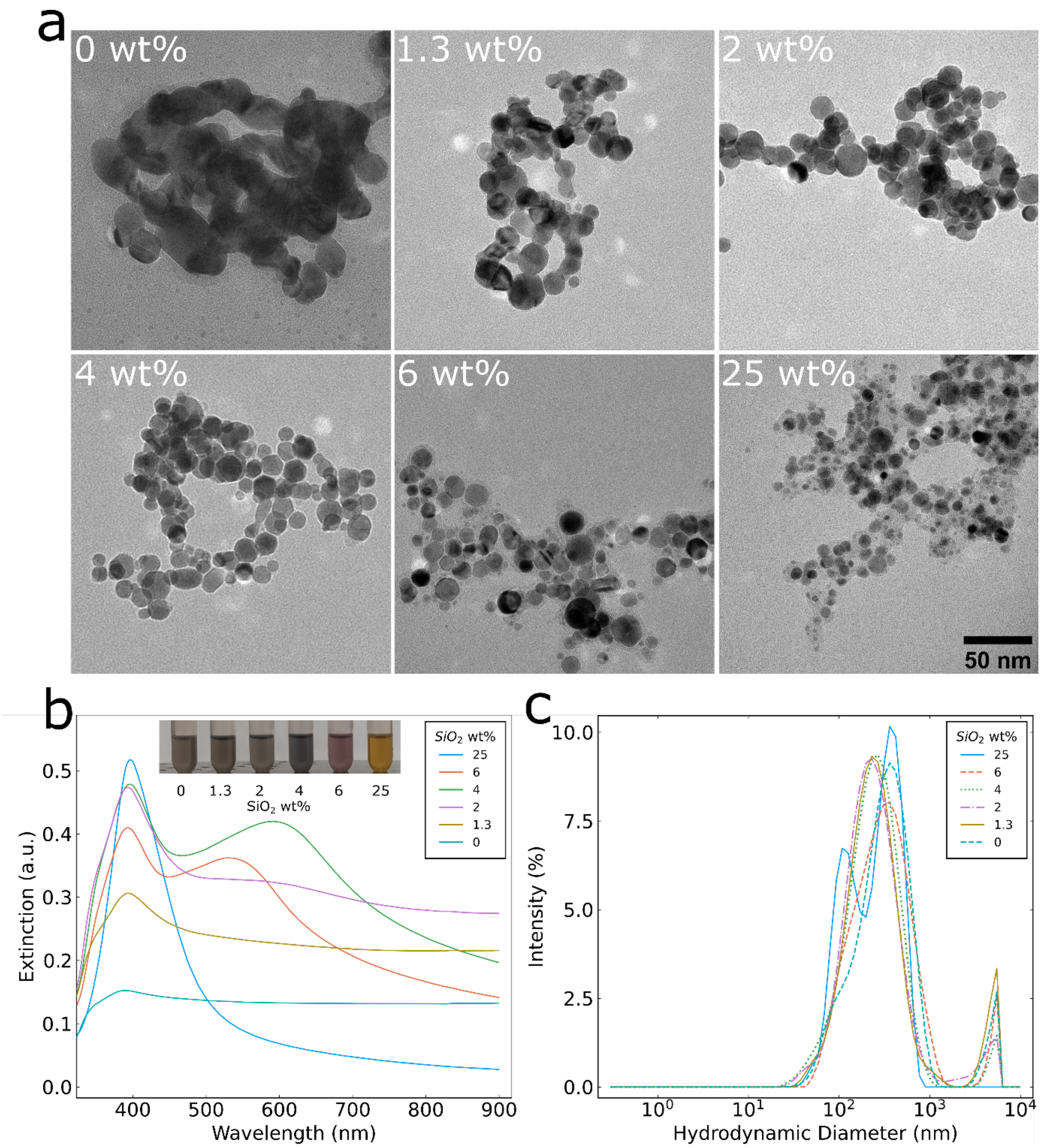

Figure S1. a) TEM images of nanoparticles retrieved from the deposited films by sonicating small fragments in ethanol. b) Extinction spectra measured from nanoparticles retrieved from deposited films by sonication, insert showing a picture taken of the suspensions in Eppendorf tubes. c) Measurements by DLS of the hydrodynamic diameter of the nanoparticle suspensions retrieved from deposited films.

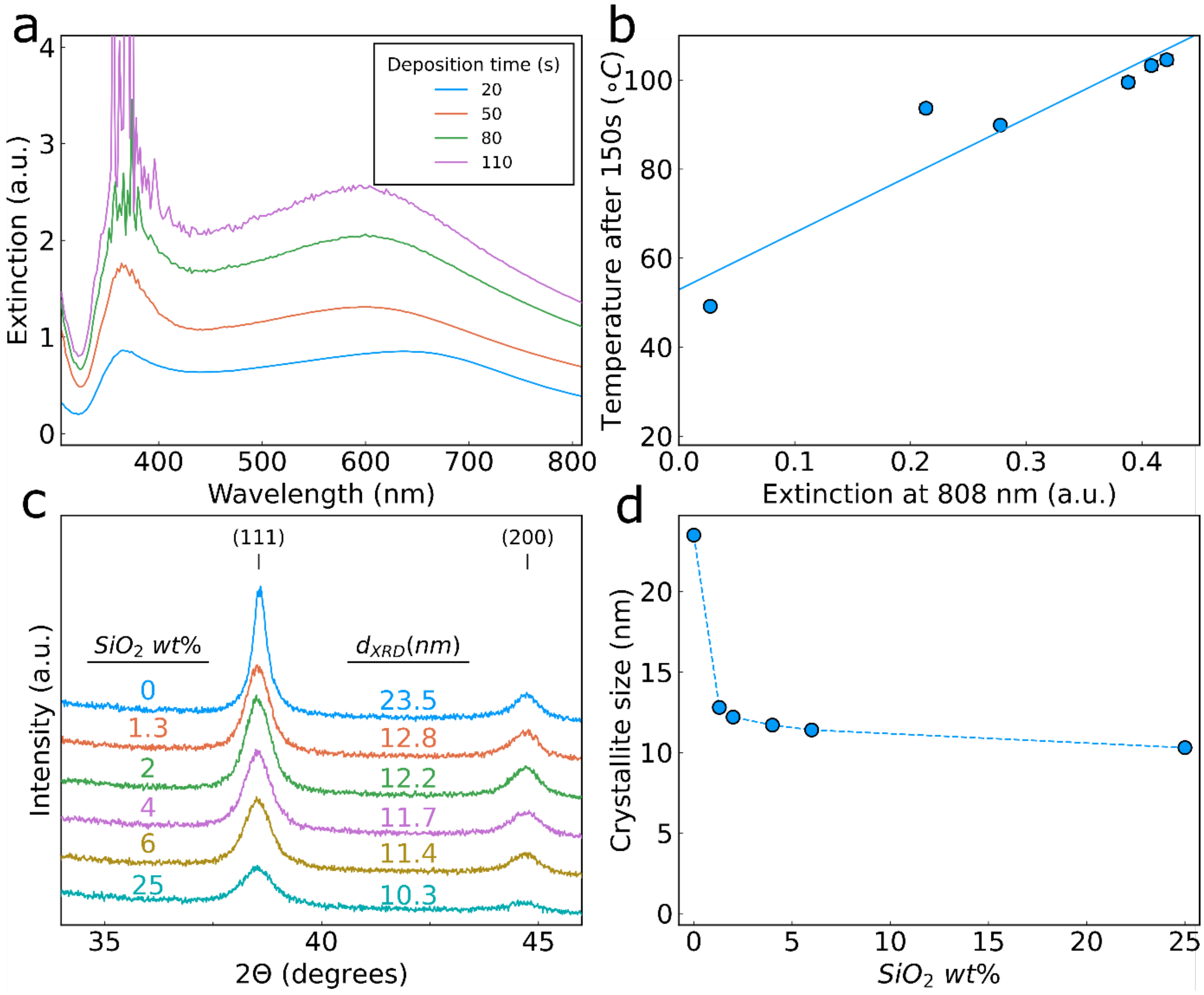

Figure S2. a) Extinction measured from films prepared with different deposition times with 2 wt% SiO_2, films synthesized for deposition times of 50, 80 and 110 s were deposited on thinner glass substrates (0.1 mm thick glass coverslips) and at 25 cm above the burner and should therefore only be loosely compared to the 20 s deposition. b) Maximum photothermal temperature reached plotted vs the extinction of the corresponding film. c) XRD patterns collected from nanoparticles deposited for 80 s onto glass substrates and subsequently scraped off for measurement. d) crystallite size determined from the XRDs in b by application of the Scherrer method to the Ag (111) peak.

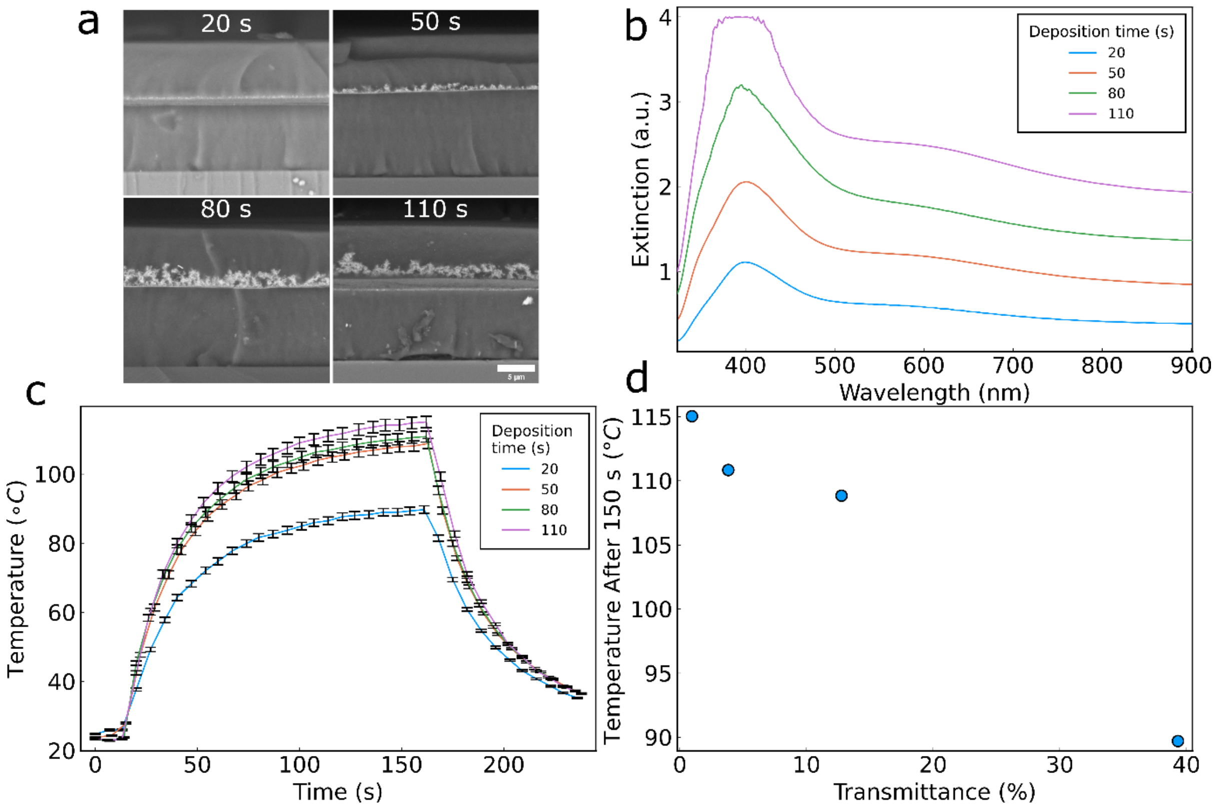

Figure S3. (a) Side-view cross sectional SEM images, (b) UV-Vis spectra, (c) thermal response of PDMS-encased Ag nanoaggregate films with 2 wt% SiO_2 content made with different deposition times and (d) Transmittance at 808 nm with respect to temperature reached after 150 s of irradiation.

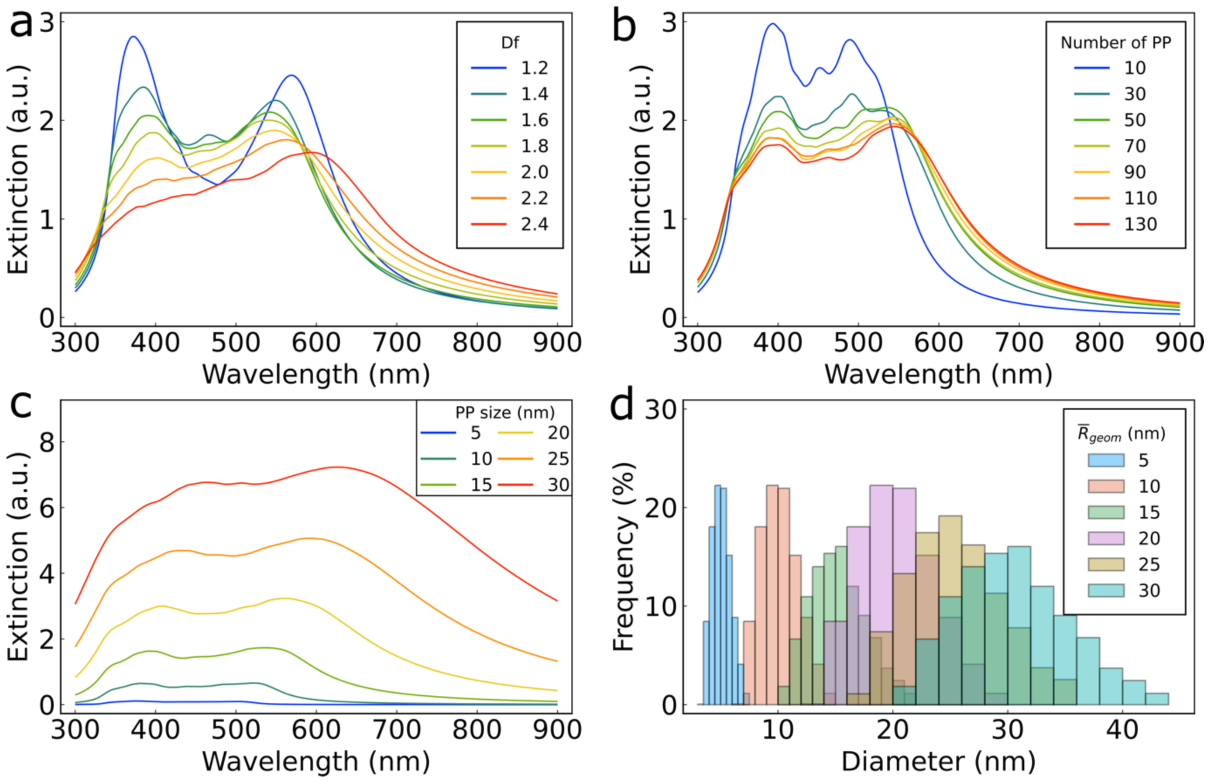

Figure S4. Unless otherwise specified the fractal dimension used was 1.8, geometric mean size of 16 nm with a geometric standard deviation of 1.2 and 100 primary particles were used per nanoaggregate. a) simulated extinction of aggregates with different fractal dimensions (Df) b) simulated extinction of aggregates containing different numbers of primary particles c) simulated extinction of aggregates containing polydisperse primary particles of different sizes d) the calculated size distributions of the polydisperse primary particles used in c).

The simulation code for aggregate generation by FracVAL and subsequent optical properties measurements is available for free in this link:

https://github.com/padmer/FracVAL_cda_helpers

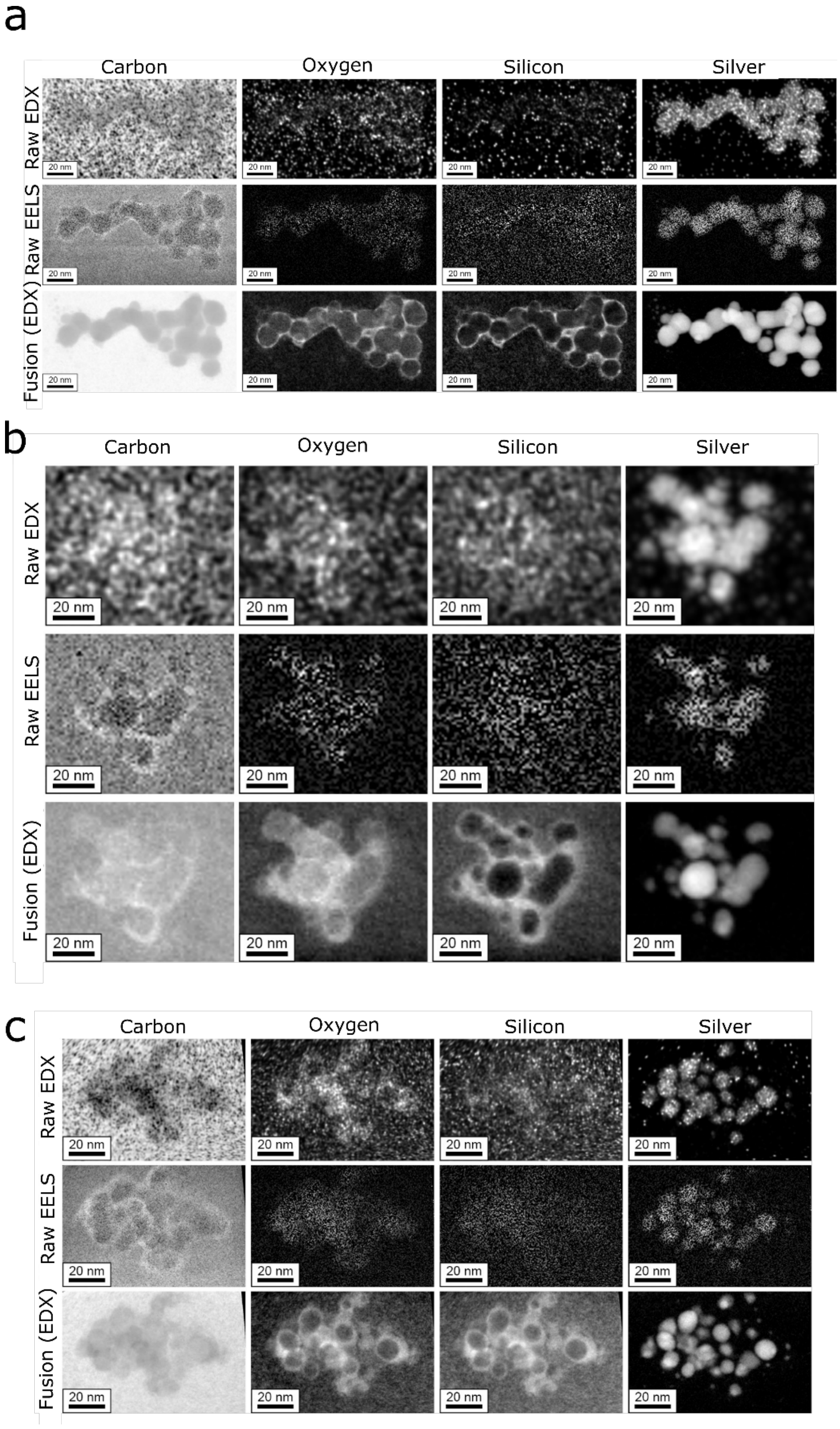

Figure S5. Raw EDX (first row) and EELS (second row) data for the detected elements used for data fusion (last row) for 2, 6 and 25 wt% SiO_2 in a, b and c respectively.

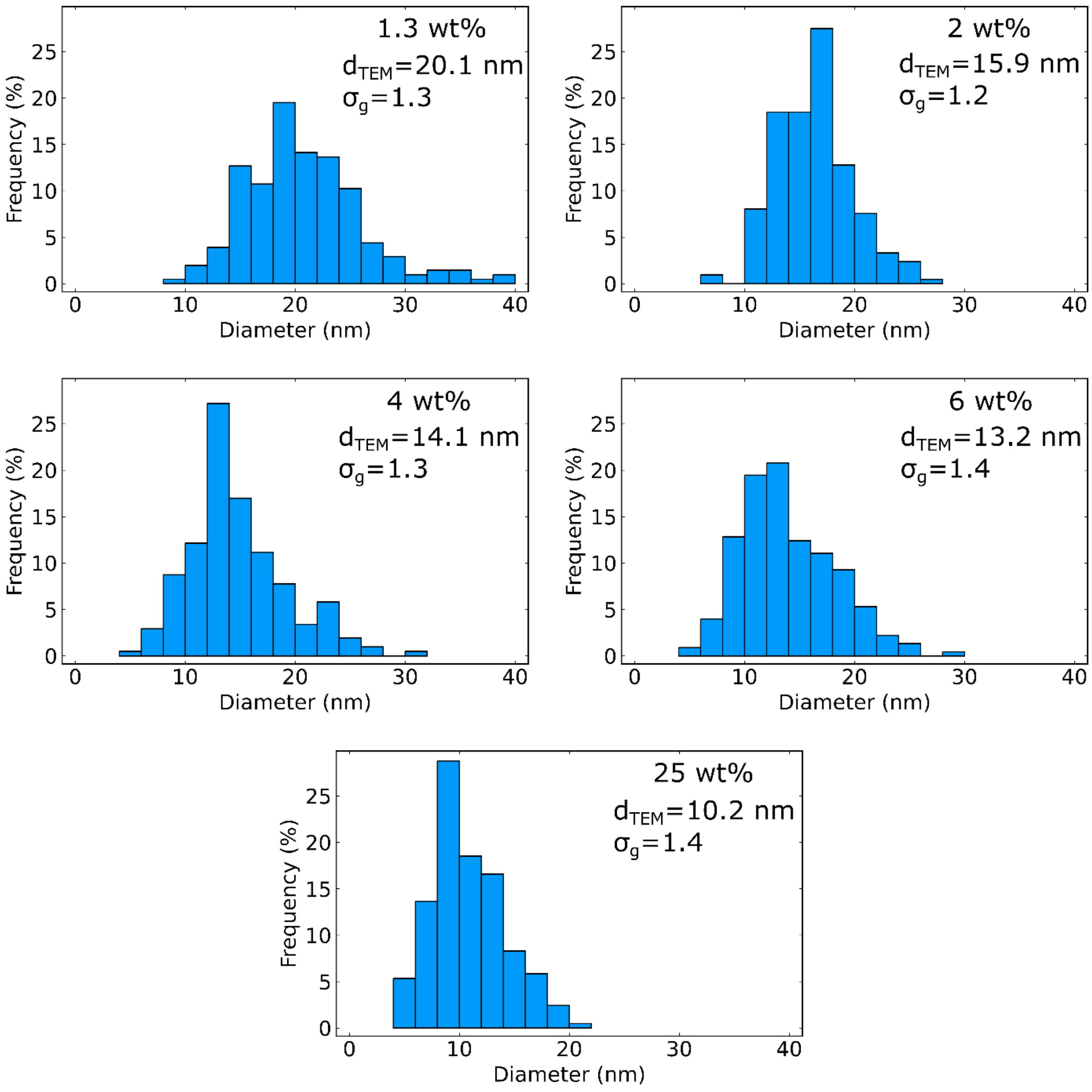

Figure S6. TEM based nanoparticle size distributions of the primary Ag nanoparticles made with varying SiO_2 contents. The geometric mean and geometric standard deviation are also shown. N >100 for all samples. Images used for counting are shown in Figure S7.

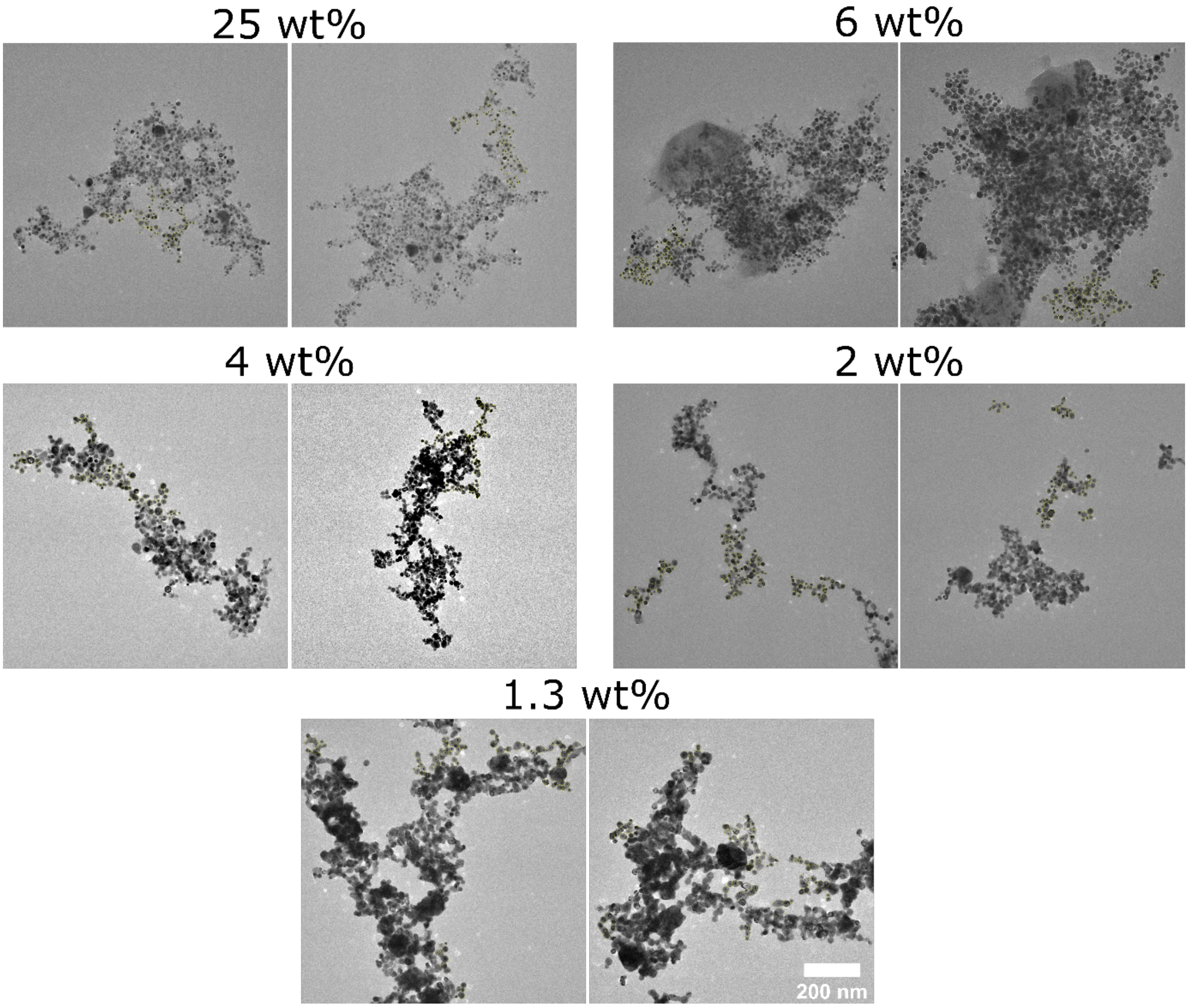

Figure S7. TEM images used for counting nanoparticle size distributions in Figure S6. Two images were counted per condition. The scale bar can be found in the bottom right image.

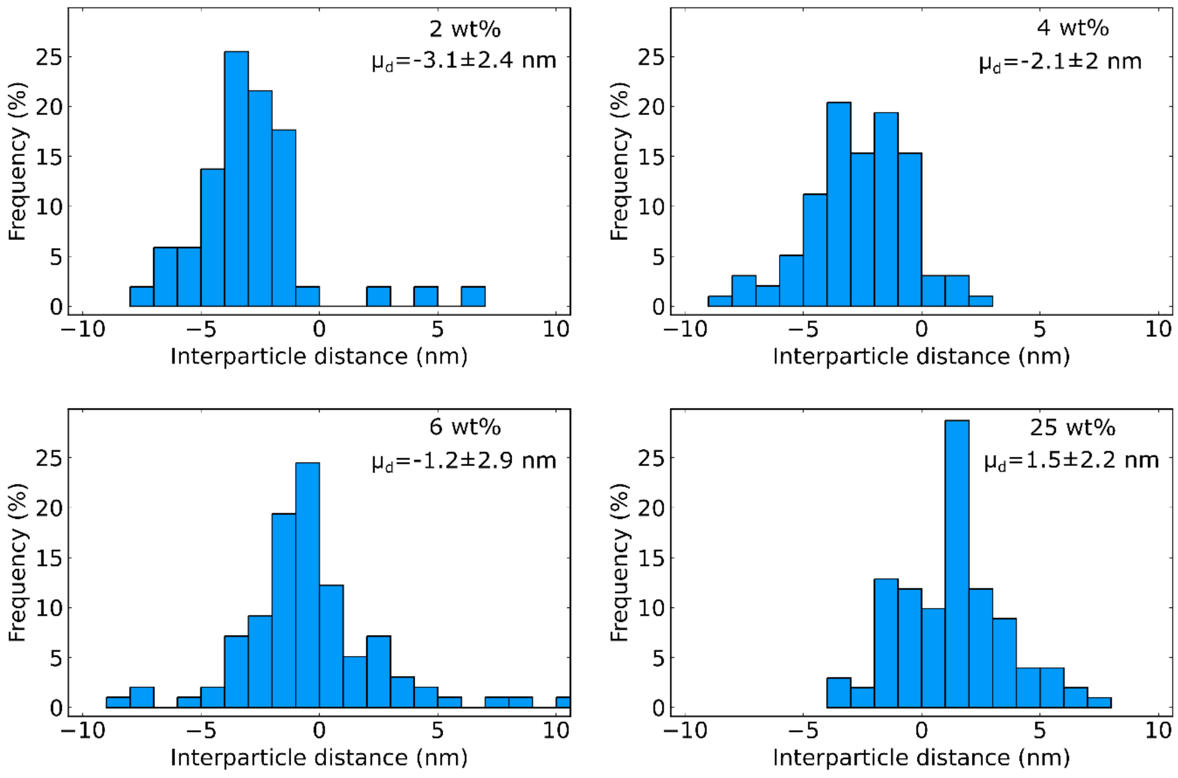

Figure S8. TEM based interparticle distances between a particle and its two closest neighbors assessed for nanoaggregates synthesized with 2, 4, 6 and 25 wt % SiO_2. Due to dense aggregates and highly overlapping structures 0 and 1.3 wt% SiO_2 were not measured. The geometric mean and standard deviation are also shown. N > 50 particles for all samples.

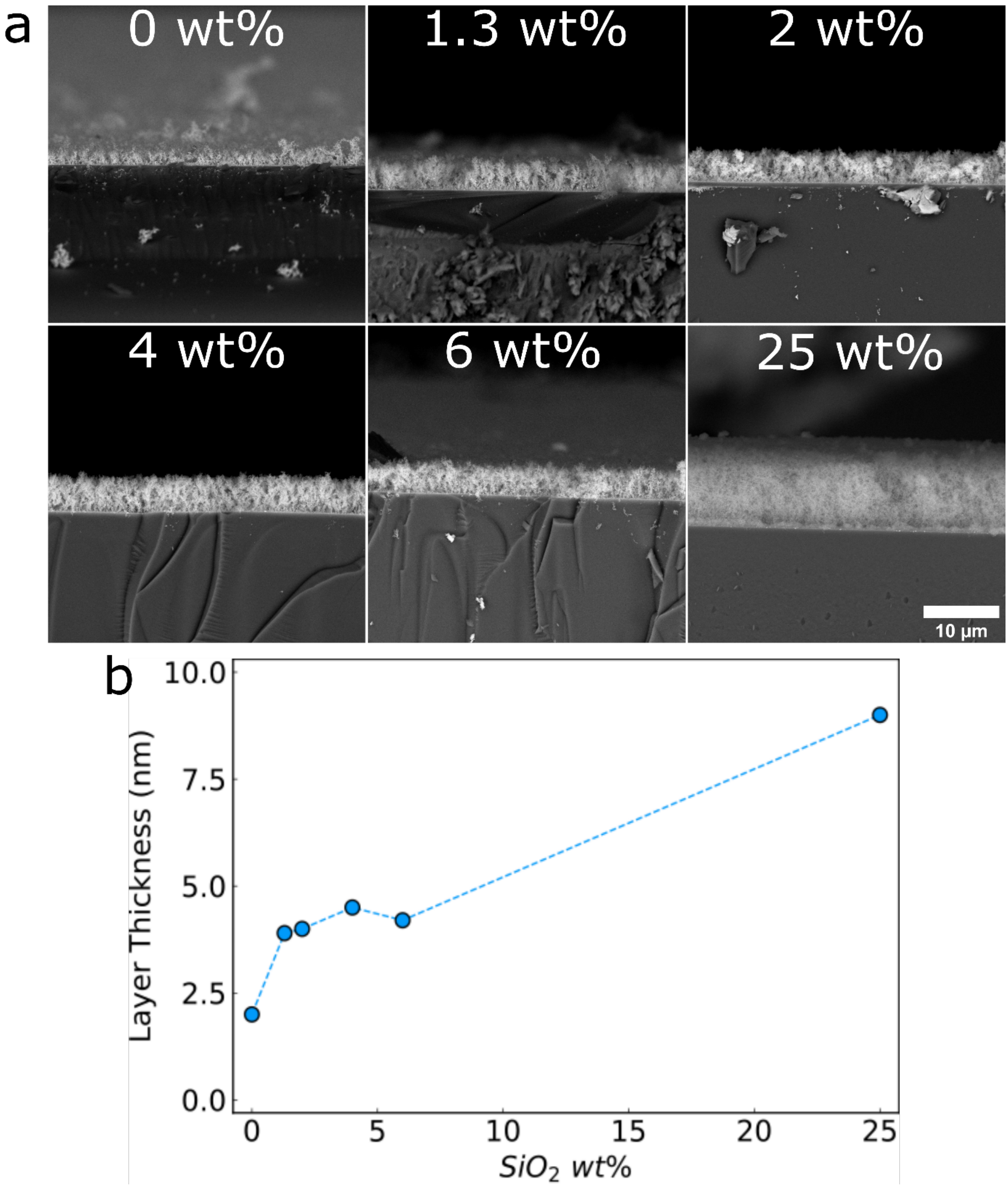

Figure S9. a) Side view images of the deposited with different SiO_2 wt% contents b) Film thicknesses measured from the images in a) plotted against the SiO_2 wt% c) SiO_2 wt% measured by EDS with the background SiO_2 contribution from the glass substrate removed by using Ca in the glass as a reference.

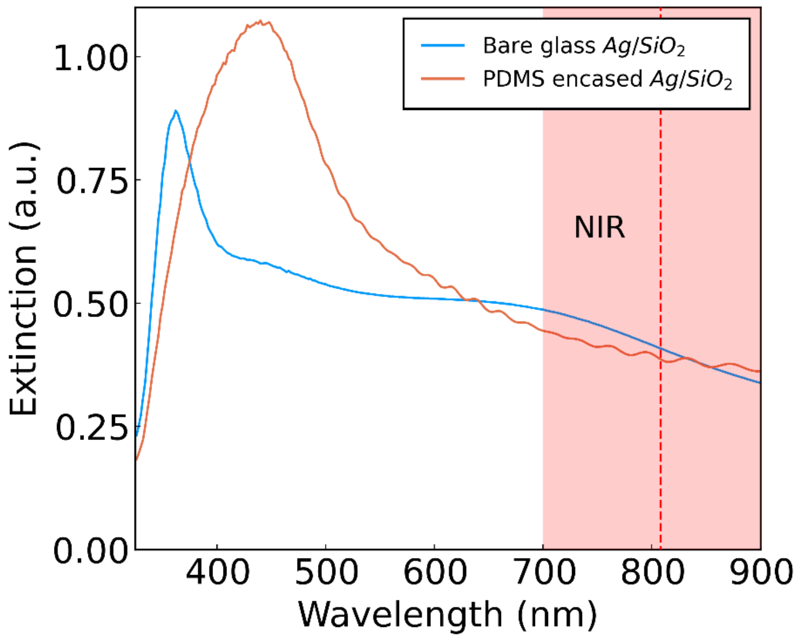

Figure S10. UV-Vis absorbance spectra of uncoated and PDMS encased films with a deposition time of 20 s.

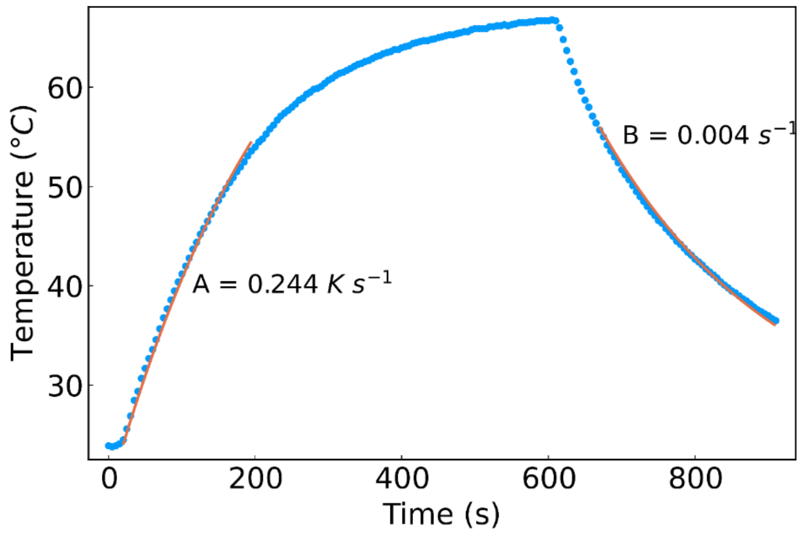

Figure S11. Temperature measurements performed with a thermocouple immersed in 300 µL PBS with one photothermal film (7x7 mm) under laser irradiation for 600 s. First the parameter B is attained from the cooling of the water and subsequently the parameter A is fitted under laser irradiation using the B parameter previously obtained following the protocol of Breitenborn et al.[38]

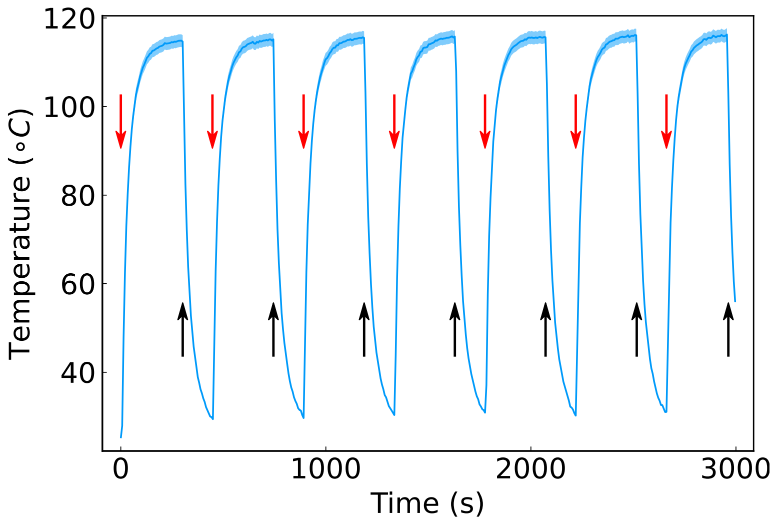

Figure S12. Thermal response of 2 wt% SiO_2 dielectric spacer Ag nanoaggregates, PDMS-encased photothermal films in air upon repeated heated (λ = 808 nm, 1 W/cm^2) and cooling. Red arrows show when the laser is switched on and black arrows when it is switched off.

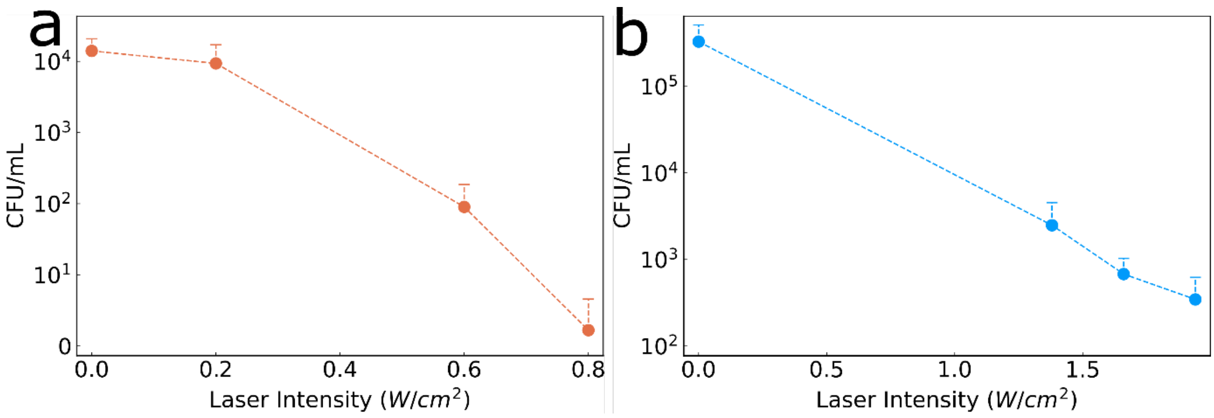

Figure S13. a) *S. aureus* CFUs retrieved from substrates irradiated for 10 minutes at different laser intensities in air. b) Colonies of *E. coli* DH5α with plasmid pJN105 GFP Hygromycin retrieved from substrates irradiated in air for 600 s with laser pulses consisting of 2 s on and 5 s off at varying laser intensities.

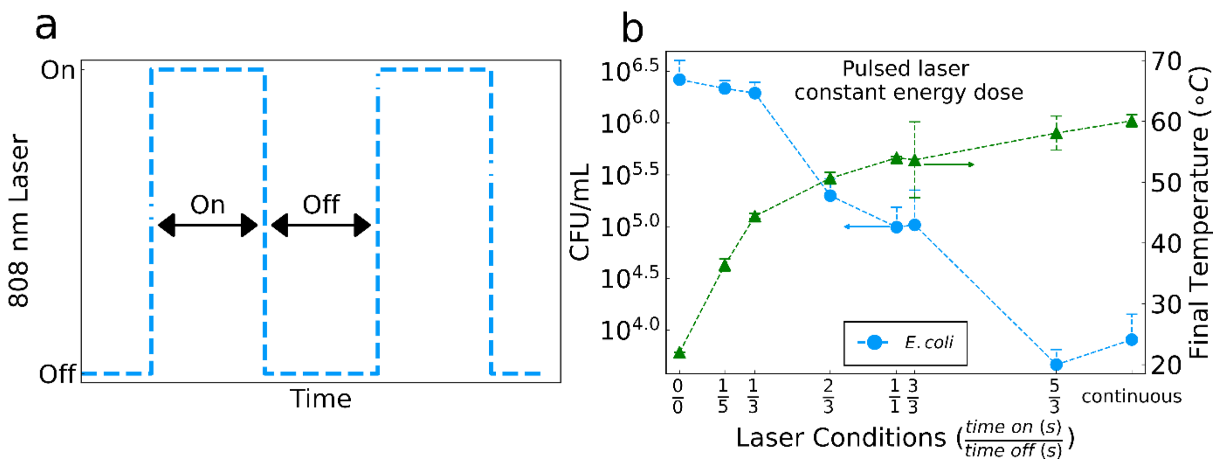

Figure S14. a) Demonstration of the pulsed irradiation conditions. (b) Bacteria quantification (CFU/mL) of *E. coli* (blue circles, left axis) and final temperature (green triangles, right axis) as a function of the pulsed laser conditions (time on/time off).

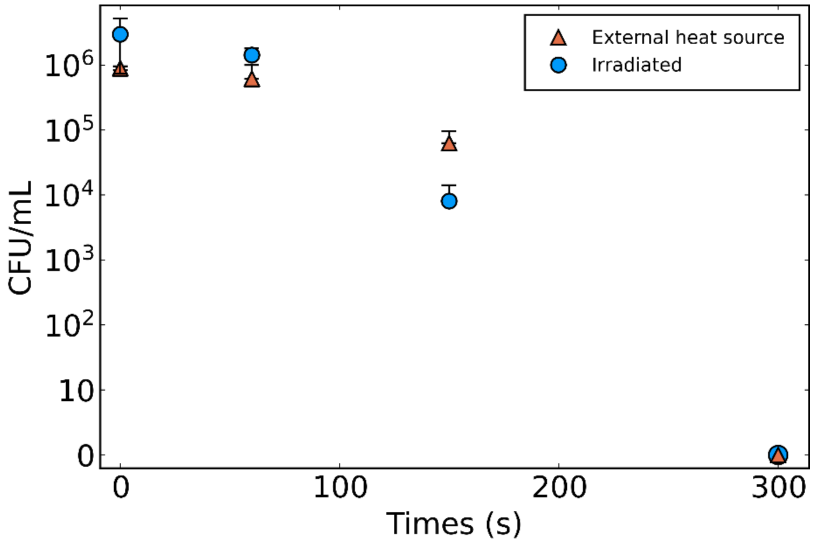

Figure S15. CFU counts of *E. coli* HVM52 retrieved from substrates immersed in liquid and heated using an external heat source (orange circles) to mimic the temperatures induced by NIR irradiation. For comparison, CFU counts retrieved after NIR irradiation and previously shown in Figure 6 are also included (blue circles). Each circle represents the mean of three biological replicates and the error bars represent the standard deviation.

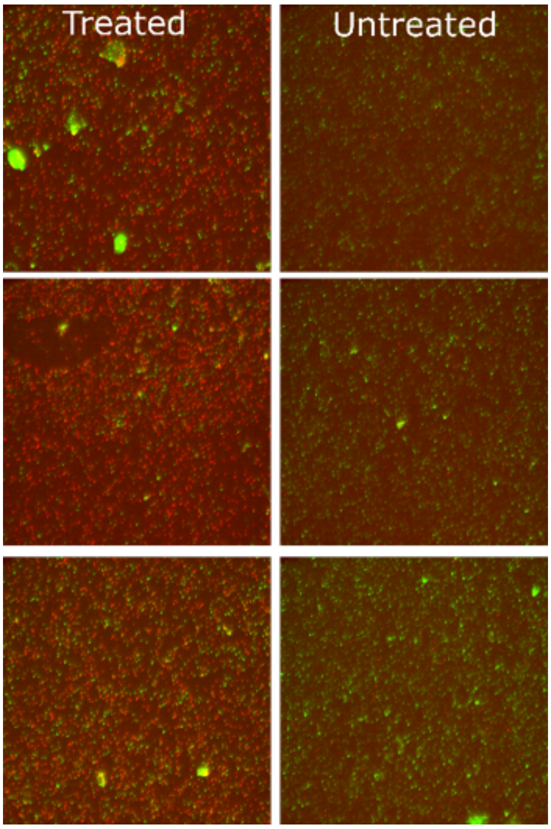

Figure S16. Microscopy images obtained with a 20x working objective of bacterial biofilm either untreated or irradiated for 600 s stained with live/dead (green/red) viability assay and acquired with the same exposure times and contrast/brightness settings.

Reference	Material	Laser wavelength and intensity	Irradiation time (s)	Pathogen	% reduction
Fang et al.[39]	$Ag@Fe_3O_4$	808 nm 2 W/cm^2	600	*E. coli & S. aureus*	100
Fan et al.[24]	Ag nano triangles	808 nm 2 W/cm^2	600	*E. faecalis*	98
Khantamat et al.[23]	Au nanoshells	810 nm 2.5 W/cm^2	600	*E. faecalis*	~100
Teng et al.[22]	Gold nanocrosses	808 nm 3 W/cm^2	300	*P. aeruginosa*	100
D'Agostino et al. [25]	Silver nanoplates	808 nm 0.26 W/cm^2	1200	*E. coli & S. aureus*	100

Table S1. Comparison of different antibacterial plasmonic photothermal nanomaterials comparing the laser wavelength, intensity and irradiation time used and the effect it has on the studied bacteria

Details of data fusion model applied to EDX/EELS data

The datacubes were treated using the block-weighted data fusion workflow described by Thersleff et al. First, outliers and spectral artefacts from both EELS datacubes were removed and the zero-loss peak was used to align both datasets in energy. Subsequently, the datacubes were normalized by their total variance and linked together along with EDX using low-level data fusion. Prior to linking, the low-loss and core-loss datacubes were subjected to a predictive weighting scheme, allowing them to dominate the variance of the joint model. In this way, the compositional information from EELS is used to predict the connected compositional response in EDX. Following data fusion, the joint dataset was decomposed using principal component analysis (PCA) and the mathematical rank was estimated from manual inspection of the scree plot, loading curves, and score maps. The mathematical rank was observed to be higher than the chemical rank of the dataset, so this was used for truncation and reconstruction. Finally, the predicted EDX datablock was extracted from the joint model and scaled back to the original space. Residuals between the raw EDX data and the data fusion model showed minimal loss of chemical information, and the compositional maps were then generated from this model.

Figure S5 compares the raw EELS and EDX maps with the EDX maps derived from data fusion that were used to generate the RGB composite in the main manuscript. The raw EDX datablock in each case is very sparse due to the low collection efficiency of the EDX detector for the given dwell time, with only a few counts registered per pixel. Consequently, mapping or even performing PCA on this datablock is challenging. A common approach to deal with the high data sparsity is to employ spectral-spatial smoothness assumptions by locally averaging neighboring spatial pixels and energy channels through the application of a 3D Gaussian kernel. However, this degrades the image resolution while failing to recover a meaningful morphology in all of these elemental maps aside from the Ag. In contrast, the EELS datablock exhibits a high signal to noise ratio by virtue of its high collection efficiency.

However, the ionization edges of greatest interest have a low signal to background ratio and require sophisticated modelling. This is particularly evident with Si-K, as this edge requires extrapolation of both the underlying plasmon peak as well as the overlapping Ag N2,3 edges. This is complicated by the low dwell time necessary for the low-loss datacube used to acquire this edge, which is a consequence of the very large dynamic range required to prevent saturation of the camera on the zero-loss peak. Consequently, the Si map is characterized by systematic errors and appears noisy. Data fusion solves both of these problems by first extending the spectral range of EELS with the EDX datablock and then combining the high signal to noise ratio of EELS with the high signal to background ratio of EDX. This is permitted by first extracting statistically significant features corresponding to the compositional variations in the EELS data block. Since both EELS and EDX spectra are collected simultaneously and, thus, originate from the same sample volume, these features are directly correlated to the compositional variance captured within the EDX datablock, providing a physical justification for estimating the missing values without the need for employing a blurring kernel. The resulting EDX model can then be used to estimate the compositional maps using standard EDX mapping procedures.
